# SARS-CoV-2 Evolution and Spike-Specific CD4+ T-Cell Response in Persistent COVID-19 with Severe HIV Immune Suppression

**DOI:** 10.3390/microorganisms10010143

**Published:** 2022-01-11

**Authors:** Hortensia Álvarez, Ezequiel Ruiz-Mateos, Pedro Miguel Juiz-González, Joana Vitallé, Irene Viéitez, María del Carmen Vázquez-Friol, Isabel Torres-Beceiro, Alberto Pérez-Gómez, Pilar Gallego-García, Nuria Estévez-Gómez, Loretta De Chiara, Eva Poveda, David Posada, Josep M. Llibre

**Affiliations:** 1 Infectious Diseases Unit, Department of Internal Medicine, University Hospital of Ferrol, SERGAS, 15405 Ferrol, Spain; 2 Clinic Unit of Infectious Diseases, Microbiology and Preventive Medicine, Institute of Biomedicine of Seville, IBiS, Virgen del Rocío University Hospital/CSIC/University of Seville, 41013 Seville, Spain; ezequiel.ruizmateos@gmail.com (E.R.-M.); jvitalle001@gmail.com (J.V.); alberto.pergom@gmail.com (A.P.-G.); 3 Microbiology Department, University Hospital of Ferrol, SERGAS, 15405 Ferrol, Spain; pmjuizgonzalez@gmail.com (P.M.J.-G.); isabel.torres.beceiro@sergas.es (I.T.-B.); 4 Group of Virology and Pathogenesis, Galicia Sur Health Research Institute (IIS Galicia Sur), Complexo Hospitalario Universitario de Vigo, SERGAS-UVIGO, 36213 Vigo, Spain; irene.vieitez@iisgaliciasur.es (I.V.); eva.poveda.lopez@sergas.es (E.P.); 5 Internal Medicine Department, University Hospital of Ferrol, SERGAS, 15405 Ferrol, Spain; maria.del.carmen.vazquez.friol@sergas.es; 6 CINBIO, Universidade de Vigo, 36310 Vigo, Spain; gpilargg@gmail.com (P.G.-G.); nuestevez@uvigo.es (N.E.-G.); ldechiara@uvigo.es (L.D.C.); dposada@uvigo.es (D.P.); 7 Galicia Sur Health Research Institute (IIS Galicia Sur), SERGAS-UVIGO, 36213 Vigo, Spain; 8 Infectious Diseases and “Fight AIDS and Infectious Diseases” Foundation, University Hospital Germans Trias i Pujol, 08916 Badalona, Spain; jmllibre@flsida.org

**Keywords:** SARS-CoV-2, HIV, CD4+ T cell response

## Abstract

Intra-host evolution of severe acute respiratory syndrome coronavirus 2 (SARS-CoV-2) has been reported in cases with persistent coronavirus disease 2019 (COVID-19). In this study, we describe a severely immunosuppressed individual with HIV-1/SARS-CoV-2 coinfection with a long-term course of SARS-CoV-2 infection. A 28-year-old man was diagnosed with HIV-1 infection (CD4+ count: 3 cells/µL nd 563000 HIV-1 RNA copies/mL) and simultaneous *Pneumocystis jirovecii* pneumonia, disseminated *Mycobacterium avium complex* infection and SARS-CoV-2 infection. SARS-CoV-2 real-time reverse transcription polymerase chain reaction positivity from nasopharyngeal samples was prolonged for 15 weeks. SARS-CoV-2 was identified as variant Alpha (PANGO lineage B.1.1.7) with mutation S:E484K. Spike-specific T-cell response was similar to HIV-negative controls although enriched in IL-2, and showed disproportionately increased immunological exhaustion marker levels. Despite persistent SARS-CoV-2 infection, adaptive intra-host SARS-CoV-2 evolution, was not identified. Spike-specific T-cell response protected against a severe COVID-19 outcome and the increased immunological exhaustion marker levels might have favoured SARS-CoV-2 persistence.

## 1. Introduction

There is evidence of intra-host evolution of severe acute respiratory syndrome coronavirus 2 (SARS-CoV-2) in cases with persistent coronavirus disease 2019 (COVID-19), including severely immunosuppressed HIV- and SARS-CoV-2-coinfected individuals [1]. Here, we report the study of a severely immunosuppressed HIV-1 individual with a long-term course of SARS-CoV-2 infection, and an unexpectedly favourable clinical outcome. Likewise, we analyse the spike-specific CD4+ T-cell response and the intra-host evolution of SARS-CoV-2, to better understand why the clinical course and prognosis were so favourable despite the high-risk situation.

## 2. Materials and Methods

### 2.1. Severe Acute Respiratory Syndrome Coronavirus 2 (SARS-CoV-2) Plasma RNA

SARS-CoV-2 RNA was measured in plasma samples at several time points: week 4 and week 12 after the first positive SARS-CoV-2 real-time reverse transcription polymerase chain reaction (rRT-PCR).

#### 2.1.1. SARS-CoV-2 RNA Extraction in Plasma

Viral RNA was extracted from 500 µL plasma samples using the QIAmp viral RNA mini kit (QIAGEN, Hilden, Germany) and the automatized QIAcube system (QIAGEN, Hilden, Germany), and was eluted in 50 µL of buffer following the manufacturer’s instructions. The positive (SARS-CoV-2 Standard, EDx Exact Diagnostics) and negative (SARS-CoV-2 Negative, EDx Exact Diagnostics) controls were also extracted using the same procedure.

#### 2.1.2. Droplet Digital Polymerase Chain Reaction (PCR) Analysis

SARS-CoV-2 viral load was quantified by reverse transcriptase droplet digital PCR (RT-ddPCR), including the one-step reverse transcription (One-Step RT-ddPCR Advanced Kit for Probes, Bio-Rad Laboratories, Hercules, CA, USA) and the triplex probe assay for PCR amplification (2019-nCoV CDC ddPCR Triplex Probe Assay, Bio-Rad Laboratories). The assay contains primers and probes targeting two regions of the SARS-CoV-2 nucleocapsid gene (N1 and N2) and the human RNase P gene (*RPP30*) as internal control. The reaction mixture was performed with 5.5 µL of SARS-CoV-2 RNA sample and following the manufacturer’s instructions. All the samples were tested in duplicate. Data analysis was performed using the QuantaSoft Analysis Pro Software (Bio-Rad Laboratories) which showed the results as copies per microliter of 1x ddPCR reaction. All viral load values were recalculated to copies per millilitre of plasma, and the sensitivity threshold was 100 copies/mL.

### 2.2. SARS-CoV-2 Genomic Analysis from Nasopharyngeal Samples

Genomic sequencing from nasopharyngeal samples was successful at three time points after the first positive SARS-CoV-2 rRT-PCR, at 44 (first time point, T1), 61 (second time point, T2) and 65 (third time point, T3) days.

#### 2.2.1. Complementary DNA (cDNA) Synthesis and Multiplex Amplification

We followed the ARTIC sequencing protocol (v.3) [2] with slight modifications. We retrotranscribed 11 µL of RNA from each sample to cDNA using the SuperScript IV reverse transcriptase (Invitrogen, Waltham, MA, USA). Multiplex PCR was performed using the ARTIC primer Pool1 and Pool2 (IDT, Coralville, IA, USA) and the Q5 Hot Start DNA polymerase (New England Biolabs, Ipswich, MA, USA). Next, we mixed the corresponding PCR products before cleaning (1.2:1 ratio beads to sample). We quantified the clean PCR products with the Qubit 3.0 using the dsDNA HS or BR kit (Thermo Fisher Scientific, Waltham, MA, USA) and checked amplicon size with the 2200 TapeStation D1000 kit (Agilent Technologies, Santa Clara, CA, USA). To obtain an adequate concentration of the PCR product for library preparation, we used two different protocols: (A) 2.5 µL of cDNA, 98 °C for denaturation and 30 PCR cycles, and (B) 5 µL of cDNA, 95 °C for denaturation and 35 PCR cycles. For sample 2522 we produced two replicates using 30 PCR cycles (2522.A1 and 2522.A2). For sample 2651 we obtained two replicates using 30 PCR cycles (2651.A1 and 2651.A2). To increase the DNA input for library preparation we produced a third replicate using 35 PCR cycles (2651.B1). For sample 2652 the 30 cycles protocol did not render enough PCR concentration, therefore we carried out two replicates using the 35-cycle protocol (2652.B1 and 2652.B2). Since sample 2858 was negative for SARS-CoV-2, we only obtained a single replicate using 35 PCR cycles (2858.B). For the plasma samples 2879, 2880 and 2881, we performed a nested-PCR using the 35 PCR cycle protocol. A single replicate was obtained for these samples (2879.B, 2880.B and 2881.B).

#### 2.2.2. Library Construction and Genome Sequencing

We built 11 whole-genome sequencing libraries employing the DNA Prep (M) Tagmentation kit (Illumina, San Diego, CA, USA) using ¼ of the recommended volume, with an average of 152 ng of input DNA. Finally, we checked the size of the libraries and quantified them as described above. We sequenced the libraries on an Illumina MiniSeq (PE150 reads) at the sequencing facility of the University of Vigo.

#### 2.2.3. Mutation Calling

We aligned the reads to the reference SARS-CoV-2 sequence from isolate Wuhan-Hu-1 (MN908947.3) using BWA-mem [3] trimmed using iVar [4] and evaluated their quality using Picard v.2.21.8 [5]. Depth of coverage along the genome was calculated using SAMtools depth v.1.10 [6]. To identify mutations (single nucleotide variants (SNVs) and indels) we used iVar, with a minimum base quality threshold of 20, a minimum read depth of 10 and a minimum allele frequency threshold of 0.03. We built the consensus sequences using iVar consensus, with a minimum variant allele frequency (VAF) threshold of 0.5 and a minimum depth of 10 reads, assigning them to a clade with Nextclade (https://clades.nextstrain.org, accessed on 19 October 2021) and to a lineage using Pangolin [7].

### 2.3. T-Cell Response Analysis

The cellular immune response was analysed at three time points: at 20 days before the first positive SARS-CoV-2 rRT-PCR (baseline) and after the first positive SARS-CoV-2 rRT-PCR, at first month (M1) and third month (M3).

#### 2.3.1. Cell Stimulation

Peripheral blood mononuclear cells (PBMCs) were thawed, washed and rested for 1 h in DNase I (Roche Diagnostics, Indianapolis, IN, USA)-containing R-10 complete medium (RPMI 1640 supplemented with 10% heat-inactivated fetal bovine serum, 100 U/mL penicillin G, 100 l/mL streptomycin sulfate, and 1.7 mM L-glutamine). 1.5 × 10^6^ PBMCs were stimulated in vitro for 6 h with overlapping protein S (PepMix™ SARS-CoV-2; Spike Glycoprotein, from JPT, Berlin, Germany). 1.5 × 10^6^ PBMCs incubated with the proportional amount of DMSO were included in each batch of experiments as a negative control. The stimulation was performed in the presence of 10 µg/mL of brefeldin A (Sigma Chemical Co, St. Louis, MO, USA), and 0.7 µg/mL of monensin (BD Biosciences), anti-CD107a-BV650 (clone H4A3; BD Biosciences, USA) monoclonal antibody and unconjugated CD28 and CD49d as previously described [8]. T-cell specific response was defined as the frequency of cells with detectable intracellular cytokine production, after background subtraction of the unstimulated condition. For this analysis 1 × 10^6^ events were acquired and a median of 4.72 × 10^5^ live T-cells were gated.

#### 2.3.2. Immunophenotyping and Intracellular Cytokine Staining

After culture, PBMCs were centrifuged, pelleted and washed with Phosphate-buffered saline (PBS) and incubated 35 min at room temperature with LIVE/DEAD Fixable Aqua Dead Cell Stain (Life Technologies), anti-CD14-BV510 (clone MφP9), anti-CD19-BV510 (clone SJ25C1), anti-CD56-BV510 (clone NMCAM16.2), anti-CD45RA-FITC (clone L48), anti-CD8-APC (clone SK-1), anti-CD27-APCH7 (clone M-T271), anti-PD-1-BV786 (CD279, clone EH12-1), and anti-CD3-BV711 (clone SK-7) (BD Bioscience); and anti-TIGIT-PerCPCy5.5 (clone A15153G) (BioLegend). Then PBMCs were permeabilized using the Cytofix/cytoperm BD kit (Cat:554714, Bioscience) (eBioscience™) following the manufacturers’ protocol and intracellularly stained at 4 °C with anti-IL-2-BV421 (clone MQ1-17H12), anti-IFN-γ-PE-Cy7 (clone B27) (BD Bioscience), anti-TNF-α-AF700 (clone Mab11) (BD Pharmingen), anti-Perforin-PE (clone B-D48) (BioLegend) Perforin. T-cells were gated based on CD3 and CD8 expression. Each subset (Total Memory, MEM; Central Memory, CM; Effector Memory, EM; and terminally differentiated effector memory, TEMRA) was gated based on CD45RA and CD27 expression. Flow cytometry analyses were performed on an LRS Fortessa flow cytometer using FACS Diva software (BD Biosciences). Data were analyzed using the FlowJo software (Treestar, Ashland, OR, USA). At least 1 × 10^6^ events were acquired per sample.

## 3. Results

### 3.1. Case Report

In December 2020, a 28-year-old man was admitted to hospital with fever, night sweats, asthenia, weight loss and diarrhea, in the last 2 months. He had known his HIV-1 serostatus 3 weeks before admission but he had not been able to access the HIV care system.

On physical examination, he was cachectic, had oral thrush and oral villous leukoplakia, without other remarkable findings. Blood tests disclosed microcytic anaemia (hemoglobin 9.8 g/dL, mean corpuscular volume 74 femtolitres) and hypoalbuminemia. CD4+ T-cell count was 3 cells/µL and plasma HIV-1 RNA 563,000 copies/mL. A computerized tomography (CT) scan showed a micronodular lung pattern, supraclavicular, mediastinal and retroperitoneal lymphadenopathies, as well as pleural, pericardial and peritoneal effusions. Bronchoalveolar lavage samples were obtained through bronchoscopy and empiric treatment of tuberculosis and *Mycobacterium avium complex* (MAC) was started with isoniazid, rifampin, pyrazinamide, ethambutol and azithromycin. *Pneumocystis jirovecii* (*P. jirovecii*) DNA was detected by PCR in a bronchoalveolar lavage sample and trimethoprim-sulfamethoxazole (TMP-SMX) and steroids were initiated. MAC grew from bronchoalveolar lavage, blood, urine and stool cultures. Treatment was streamlined to rifampin, ethambutol and azithromycin.

An antiretroviral therapy (ART) based on tenofovir disoproxil fumarate/emtricitabine plus raltegravir was started 1 week after initiating treatment aimed at *P. jirovecii* and MAC. Genotypic tests revealed a non-B HIV subtype, variant F1, with 106 V > I substitution at the reverse transcriptase (RT). Once HLA-B*5701 was known, ART was streamlined to dolutegravir/lamivudine/abacavir. In addition, rifampin was switched to rifabutin, allowing dolutegravir once daily in a fixed-dose combination regimen.

Ten days after starting ART, SARS-CoV-2 was detected by rRT-PCR from a nasopharyngeal swab, in the setting of epidemiological investigations of a nosocomial outbreak contact tracing. The cycle threshold (Ct) value in rRT-PCR for ORF1ab and S genes was 10. The patient had neither hypoxemia nor overt SARS-CoV-2-related clinical manifestations over the course of hospital admission; therefore, he did not receive specific treatment against SARS-CoV-2.

Five weeks after starting ART, fever and abdominal pain appeared, anaemia got worse, inflammatory biomarkers (erythrocyte sedimentation rate, C-reactive protein, ferritin and D-dimer) strikingly increased and immature erythroid and myeloid cells were identified in peripheral blood, suggestive of a leucoerythroblastic reaction. In addition, maculopapular skin lesions appeared in a parallel fashion to tapering down of steroids, suggesting a TMP-SMX hypersensitivity reaction; so TMP-SMX was replaced by pentamidine. In a new CT scan, a micronodular lung pattern as well as pleural and pericardial effusions had disappeared and lymphadenopathies had decreased in size. New infections were ruled out. Plasma HIV-1 RNA decreased 2·0 log_10_ copies/mL and CD4+ T-cell count increased to 29 cells/µL. The exaggerated inflammatory response, along with the short interval between the initiation of opportunistic infections treatment and ART (one week), suggested an immune reconstitution inflammatory syndrome (IRIS). Thus, steroids were restarted (prednisone 1 mg/kg/day) setting a tapering schedule subsequently. The patient’s clinical condition improved and he was discharged on day 26 after the first positive SARS-CoV-2 rRT-PCR and day 36 after ART initiation. The ORF1ab and S genes Ct value remained at 16.

Between days 27 and 43 after the first positive SARS-CoV-2 rRT-PCR, the patient quarantined alone at home without any potential re-exposure to SARS-CoV-2 infection risk settings. He was readmitted due to vomiting on day 44. SARS-CoV-2 rRT-PCR Ct remained at 15 (dual target to RNA-dependent RNA polymerase-RdRp and N genes). Acute-phase reactants remained increased. Since hyponatremia was detected, a schedule of steroids was restarted and aimed at either a probable primary adrenal insufficiency in the setting of MAC adrenalitis and an IRIS: prednisone 1 mg/kg/day (equivalent dose of 9.6 mg of dexamethasone and 240 mg of hydrocortisone).

**Figure 1:**
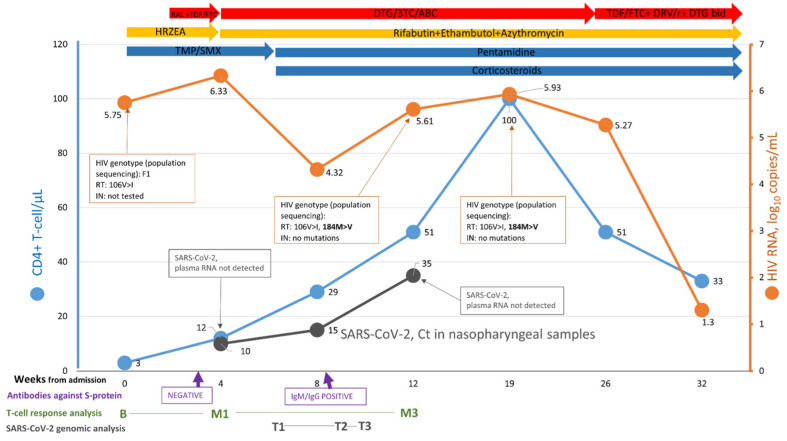
Timeline—RAL, raltegravir; TDF, tenofovir disoproxil fumarate; DTG, dolutegravir; ABC, abacavir; 3TC, lamivudine; FTC, emtricitabine; DRV/r, darunavir/ritonavir; bid, twice a day; TMP/SMX, trimethoprim/sulfamethoxazole; RT, reverse transcriptase; IN, integrase; HRZEA, isoniazid, rifampin, pyrazinamide, ethambutol, azithromycin; Ct, cycle threshold of severe acute respiratory syndrome coronavirus 2 (SARS-CoV-2) in nasopharyngeal samples. Time points of T-cell response analysis: B, Baseline; M1, Month 1; M3, Month 3. Time points of SARS-CoV-2 genomic analysis from nasopharyngeal samples: T1, time point 1; T2, time point 2; T3, time point 3. Time points represent estimations through the timeline.

The dynamics of SARS-CoV-2 RNA decay kinetics was very slow, suggesting persistent high viral loads, achieving Ct value > 35 eventually on day 66, and negative rRT-PCR after 15 weeks (day 103) (confirmed at a second sample on day 120). Antibodies against the spike protein (positive IgM and titer of IgG at 28.2 arbitrary units (AU)/mL), were detected on day 58, indicating seroconversion from a previous negative assay on day 19 (Figure 1). Neutralising antibody responses were not measured.

Eleven weeks after starting ART and nine weeks after the first detection of SARS-CoV-2 from nasopharyngeal sample, the patient achieved a satisfactory general condition, acute-phase reactants decreased, hyponatremia was solved and he was definitely discharged from hospital.

### 3.2. Human Immunodeficiency Virus (HIV) Genomic Analysis

After 20 weeks of ART with dolutegravir/lamivudine/abacavir, plasma HIV-RNA remained above 5 log_10_ copies/mL. The 184 M > V RT-mutation, associated with lamivudine resistance, was detected at week 12 after ART initiation with no integrase mutations. A salvage regimen was tailored, including: tenofovir disoproxil fumarate/emtricitabine plus ritonavir-boosted darunavir plus dolutegravir 50 mg twice daily. An HIV-RNA < 20 copies/mL was achieved four weeks later.

### 3.3. SARS-CoV-2 Plasma RNA

Despite persistent SARS-CoV-2 RNA isolation from nasopharyngeal samples including very low Ct values, RNA could not be detected in plasma samples at several time points (week 4 and week 12 after the first SARS-CoV-2 rRT-PCR).

### 3.4. SARS-CoV-2 Genomic Analysis from Nasopharyngeal Samples

**Figure 2:**
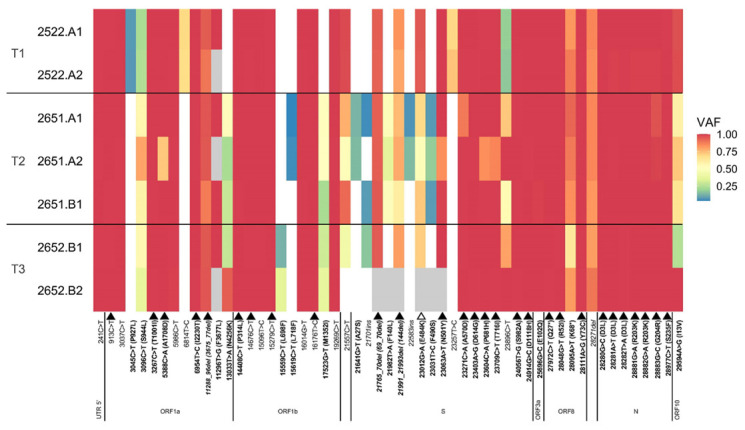
SARS-CoV-2 variant allele frequencies (VAFs) across time points, from nasopharyngeal samples. Only mutations shared by at least two replicates of the same time point are shown. The reference allele corresponding to the Wuhan-Hu-1 genomic sequence MN908947.3 is indicated in white. Missing VAF values, corresponding to positions with low coverage (<20X), are depicted in grey. On the *y*-axis, A and B indicate slightly different protocols (see Materials and Methods), while A1/A2/B1/B2 identify distinct replicates. On the *x*-axis, non-synonymous mutations are in bold and reflect the amino acid change, synonymous mutations are represented only by the nucleotide change, and insertions and deletions are in italic. Filled black triangles highlight mutations specific to the SARS-CoV-2 Alpha variant (B.1.1.7), while the open black triangle highlights the E484K mutation.

We investigated the intra-host viral evolution of SARS-CoV-2 from serial retrospective nasopharyngeal samples (time points T1, T2 and T3, as defined in materials and methods) (Figure 2).

SARS-CoV-2 was classified as an Alpha variant (Phylogenetic Assignment of Named Global Outbreak Lineages-PANGO-lineage B.1.1.7), similar to the consensus sequences of dominant contemporaneous isolates circulating by the same time, and included the relevant mutation S:E484K.

At the T1, we observed 46 mutations (four of them deletions), defined as positions with a nucleotide difference against the reference genome SARS-CoV-2 Wuhan-Hu-1 (MN908947.3), and shared by at least two replicates of the same time point (Figure 2). Thirty-six of these mutations had a VAF ≥ 0.95, suggesting they were fixed in the viral population. Except for small, most likely technical VAF fluctuations in some of the replicates, all the fixed mutations maintained a high VAF along the three time points sampled (T1-T3). The remaining 10 mutations observed at T1 had a VAF < 0.95, suggesting that they might represent intra-host mutations that were segregating in the viral population. Several of these mutations also fluctuated in VAF throughout time, but it was unclear whether changes in VAF were always reliable, due to the low coverage at some positions in some replicates. Thus, six apparently intra-host mutations were maintained at a high VAF across the three time points: 3675_3677del, 69_70del, 144del, 28095A > T (K68*), 28271del and 29594A > G (I13V), suggesting they were probably fixed, invariable mutations. Three of the intra-host mutations inferred at T1 were not detected at T2 or T3: 3045C > T (P927L), 6814T > C and 23257T > C. Of note, 3045C > T (P927L) was already at a very low VAF (0.07) in T1. Interestingly, 23012G > A (S:E484K) was already technically fixed (VAF > 0.95) at the first time point, but its frequency decreased at T2 and T3 going down to 0.49–0.72. Some intra-host mutations not detected in T1 appeared in T2 or T3: 13033T > A (ORF1a:N4256K), 15559C > T (ORF1b:L698F), 15619C > T (ORF1b:L718F), 17523G > T (ORF1b:M1352I), 21641G > T (S:A27S), 21701ins, 21982T > A (F140L), 22583ins, 23031T > C (F490S). These mutations were often detected at low VAFs, and did not reach fixation, except for 13033T > A (ORF1a:N4256K) and 23896C > T which increased through time up to very high VAFs. Most of these intra-host mutations do not seem to be very frequent worldwide (see https://outbreak.info, accessed on 19 October 2021). Only 3096C > T (ORF1a:S944L) is a bit more common, but still appears in a small number of cases (<0.5%). More than half of the intra-host mutations were located outside the spike protein and none of them corresponded with known mutations considered potentially advantageous to viral replicative capacity (see mutation list at http://covid19.datamonkey.org, accessed on 19 October 2021).

### 3.5. T-Cell Response Analysis

**Figure 3:**
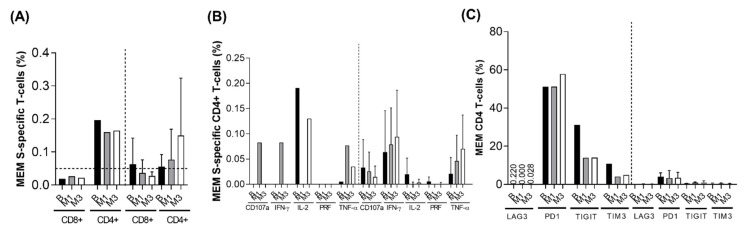
SARS-CoV-2 spike-specific T-cell response and exhaustion phenotype. (**A**) Spike-specific memory (MEM) CD4+ and CD8+ T-cell response, at baseline (B), month 1 (M1) and month 3 (M3), in our case (left panel) and control (right panel). (**B**) Cytokines produced by memory (MEM) CD4+ T-cell in response to SARS-CoV-2 S peptide, at baseline (B), month 1 (M1) and month 3 (M3): CD107a (degranulation marker), interferon-γ (IFN-γ), interleukin 2 (IL-2), perforin (PRF) and tumour necrosis factor-α (TNF-α). (**C**) Exhaustion markers from memory (MEM) CD4+ T-cells, at baseline (B), month 1 (M1) and month 3 (M3), in our case (left panel) and control (right panel): lymphocyte-activation gene 3 (LAG-3), Programmed cell death protein 1 (PD-1), T-cell immunoreceptor with Ig and ITIM (immunoreceptor tyrosine-based inhibitory motif) domains (TIGIT), T-cell immunoglobulin and mucin domain-containing protein 3 (TIM-3).

The persistent detection of SARS-CoV-2 from nasopharyngeal samples might be related to the absence of or inefficient SARS-CoV-2 specific T-cell response. We analysed the cellular immune response at three time points: baseline, M1 and M3. We observed detectable levels of Spike-specific memory (MEM) CD4+ T-cell response (Figure 3A, left panel), defined as detectable intracellular cytokine production. These levels were comparable to those found in HIV-negative SARS-CoV-2 infected subjects during acute infection (matched controls) (Figure 3A, right panel).

**Figure 4:**
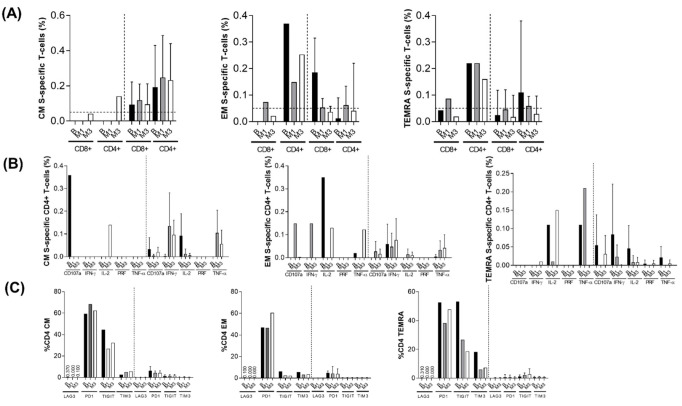
SARS-CoV-2 Spike-Specific T-Cell Response. (**A**) Spike-specific memory CD4+ T-cell subsets response, at baseline (B), month 1 (M1) and month 3 (M3), in our case (left panel) and controls (right panel): Central memory (CM), Effector memory (EM) and Subset of effector memory T-cells that expresses CD45RA (TEMRA). (**B**) Cytokines produced by memory CD4+ T-cell subsets (CM, EM, TEMRA), in response to SARS-CoV-2 S peptide, at baseline (B), month 1 (M1) and month 3 (M3) in our case (left panel) and controls (right panel): CD107a (degranulation marker), Interferon-γ (IFN-γ), Interleukin 2 (IL-2), Perforin (PRF) and Tumour necrosis factor-α (TNF-α). (**C**) Exhaustion markers from memory CD4+ T-cell subsets (CM, EM, TEMRA), at baseline (B), month 1 (M1) and month 3 (M3), in our case (left panel) and controls (right panel): Lymphocyte-activation gene 3 (LAG-3), Programmed cell death protein 1 (PD-1), T-cell immunoreceptor with Ig and ITIM (immunoreceptor tyrosine-based inhibitory).

These results were reproduced for effector memory (EM) and terminal differentiated effector memory T-cells (TEMRA), but not central memory (CM) CD4+ T-cell subsets (Figure 4A).

Spike-specific MEM CD8+ T-cell response was seen at lower levels than CD4+ T-cell response, the same happened in the control group (Figure 3A). It is important to note that this SARS-CoV-2 specific T-cell response was detectable at baseline, which strongly suggests that SARS-CoV-2 infection was indeed present some weeks before SARS-CoV-2 diagnosis (Figure 3A, left panel). We observed that, overall, interleukin 2 (IL2) was the predominant cytokine (Figure 3B), although interferon-γ (IFN-γ) and tumour necrosis factor-α (TNF-α) were produced in response to infection at one month of follow up together with the degranulation marker CD107a (Figure 3B). This response differed with HIV-uninfected controls where the predominant cytokine produced was INF-γ, although similar levels of TNF-α were observed. Similar data were found in the EM and TEMRA, but not CM subsets (Figure 4B). However, we also analysed exhaustion markers (lymphocyte-activation gene 3, LAG-3; programmed cell death protein 1, PD-1; T-cell immunoreceptor with Ig and ITIM (immunoreceptor tyrosine-based inhibitory motif) domains, TIGIT; and T-cell immunoglobulin and mucin domain-containing protein 3, TIM-3) in the bulk of the T-cell subsets. These molecules are commonly used as exhaustion markers in HIV-infection. Specifically, for instance, PD-1, TIGIT and LAG-3 contribute to HIV persistence during ART [9] and also together with TIM-3, these immune checkpoints have been associated with the translation of a competent viral reservoir in treated and untreated HIV-infection [10]. In addition, low expression of these markers has been related with the spontaneous control of HIV infection and spontaneous clearance of HCV [11]. We observed disproportionately high levels of PD1+, TIGIT+ and TIM3+ from MEM CD4+ T-cells compared with HIV-negative controls (Figure 3C). The same trend was observed for the rest of the T-cell subsets especially regarding PD-1 expression (Figure 4C). This could be associated with the extremely low levels of CD4+ T-cells rather than with SARS-CoV-2 itself.

## 4. Discussion

Data are limited regarding the evolution of SARS-CoV-2 in prolonged shedding and its impact on replicative capacity or infectious potential, particularly in patients with profound immunosuppression. Detection of SARS-CoV-2 viral load (indirectly measured by cycle threshold of PCR as a proxy or surrogate marker) is the most reliable indicator of SARS-CoV-2 persistence and contagiousness.

Some patients with cellular and humoral immune suppression including recipients of hematopoietic stem-cell transplants, cellular therapies like chimeric antigen receptor-modified (CAR) T-cell therapy [12], chemotherapy against lymphomas including anti-CD20 monoclonal antibodies [13] or acquired hypogammaglobulinemia [14], showed persistent replication and delayed SARS-CoV-2 clearance with viable SARS-CoV-2 for at least 2 months. The longest persistence of viable SARS-CoV-2 (as assessed by viral culture) has been reported in two patients with lymphoma: over 4 and 8 months, respectively [13,15]. These patients are potential persistent shedders and sources of transmission and might allow significant SARS-CoV-2 evolution. While the number of immunosuppressed individuals with available sequences remains limited, the existing data suggest that the rate of viral evolution, meaning the rate at which non-synonymous mutations lead to relevant changes in protein sequences, is accelerated within immunosuppressed individuals [16]. Thus, there is evidence of in vivo intra-host genetic evolution of SARS-CoV-2 in cases with prolonged COVID-19, such as CAR T-cell therapy recipients [17], treatment against B-cell acute lymphoblastic leukemia and lymphomas including anti-CD20 monoclonal antibody, external immunological humoral selective pressure by convalescent plasma [15,18,19,20,21], immunosuppression with antiphospholipid syndrome complicated by diffuse alveolar hemorrhage [22,23] and HIV infection [1,24,25]. Therefore, it is possible that some SARS-CoV-2 variants could have originated in patients with protracted SARS-CoV-2 infection [26]. In addition, emergence of resistance SARS-CoV-2 mutations, such as D484Y in RdRp, have been reported following failure of remdesivir treatment in an individual with B-cell immunodeficiency due to lymphocytic leukemia treated by rituximab [27].

There are discordant results regarding the clinical outcomes of COVID-19 in individuals living with well-controlled HIV under ART. Most reported cases series included low proportions of severely immunosuppressed individuals or with unsuppressed HIV viremia [28]. In a large international cohort including 286 participants, only 5.7% were not on ART and 11.3% had virological non-suppression [29]. Concomitant diagnosis with COVID-19 and tuberculosis [30], *P. jirovecii* and cytomegalovirus infection [31] and possibly IRIS [32], has been reported in HIV-1 late presenters, leading to worse COVID-19 overall outcomes. A longer course of COVID-19 disease has been reported in the setting of coinfection with HIV [33,34,35,36,37]. In a Wuhan cohort, SARS-CoV-2 rRT-PCR remained positive after a median of 30 days (IQR 20–46) in 22 HIV-coinfected individuals, achieving 77 days in one case with ART discontinuation. Duration of viral shedding was defined as the interval from symptoms onset to the two consecutive negative rRT-PCR results for SARS-CoV-2 [37]. The longest persistence of positive SARS-CoV-2 rRT-PCR in an HIV-infected patient diagnosed with B-cell lymphoma, has been reported at around 5 months [35]. Low nadir CD4+ T-cell appeared more frequently associated with longer time to viral clearance [36] and more severe outcomes [29]. In our case, findings were consistent with persistent SARS-CoV-2 infection at 103 days, defined as detection of RNA by rRT-PCR from the first nasopharyngeal sample to two consecutive negative results.

SARS-CoV-2 RNA was detected in the plasma or serum of patients with COVID-19 admitted to the intensive care unit (ICU) when neutralizing antibody response was low, and was associated with higher 28-day ICU mortality (hazard ratio, 1.84 [95%CI, 1.22–2.77] adjusted for age and sex) [38,39]. In agreement with the asymptomatic clinical course of infection in our patient, RNAemia was not detected in plasma in several time points despite the low Ct values seen from the nasopharyngeal samples.

SARS-CoV-2 variant Alpha/B.1.1.7 has been associated with higher viral loads and longer persistence. The S:E484K mutation alters antibody recognition. Since most of the intra-host mutations detected did not show a clear increase in frequency through time, at least not those located in the spike protein, we conclude that SARS-CoV-2 has not accumulated adaptive changes within this individual. Indeed, we cannot discard that some of the few observed non-Alpha non-synonymous mutations could have appeared de novo in this patient and were already fixed by selection at the first time point (day 44). However, such a rapid increase in allelic frequency seems unlikely given the pace of evolution of SARS-CoV-2 in immunosuppressed patients [14,21,23,24], and none of the non-Alpha mutations in the spike protein were already present at day 44 (and only a few outside the spike). Nonetheless, we acknowledge that the narrow temporal interval studied (44–65 days after infection) might be a limitation for detecting significant SARS-CoV-2 intra-host evolution. The lack of reproducibility across replicates for some of the low-frequency mutations suggests that some of these might be artefacts resulting from high Ct values. Interestingly, E484K was already fixed at the first time point, but waned at the second and third time points. While this reduction in frequency might reduce immune escape, it might be also a technical artefact.

There are conflicting data regarding defects in B- and T-cell function in HIV/SARS-CoV-2-coinfected patients, particularly with low CD4+ cell counts and active HIV replication, as the immune system activation is triggered by both viruses. It results in a profound lymphopenia (including both low CD4+ and CD8+ cell counts), lymphocytic activation and exhaustion, as well as increased cytokines release [28]. Diminished CD4+/CD8+ cell and Th1/Th2 cell ratios have been associated with a surge in exhausted T-cell count with simultaneous exacerbation of COVID-19-related respiratory distress [40]. Deep CD19 depletion has been unveiled in some patients with long-term and prolonged shedding of SARS-CoV-2 [24]. In addition, COVID-19 is associated with a deficit in plasmacytoid dendritic cells (pDCs) and their capacity to IFN-α production, which has been associated with disease severity and SARS-CoV-2 persistence [41,42]. Unexpectedly, in our patient, a severe CD4+ T-cell depletion, non-suppressed HIV viremia and an accumulation of life-threatening opportunistic infections, did not lead to a poorer COVID-19 course. This is in accordance with his high levels of spike-specific CD4+ T-cell response enriched in IL-2 production compared with HIV-uninfected matched controls. However, the high levels of exhaustion markers, especially PD-1 in the bulk of CD4+ T-cell subsets, enabled SARS-CoV-2 persistence despite a good quality of the response. In fact, PD-1 expression in this co-infected patient was disproportionately high, suggesting that the majority of exacerbated T-cell exhaustion was actually due to HIV-infection.

Regarding the humoral response, the level of antibodies against SARS-CoV-2 has been reported lower in individuals with non-suppressed HIV viremia [37]. In our case, antibodies against spike protein appeared at day 58 despite concomitant *Pneumocystis jirovecii* pneumonia, disseminated MAC infection and IRIS treated with steroids.

Our patient was not only unable to achieve HIV virological suppression, but rather HIV-RNA remained above 5 log_10_ copies/mL under ART at week 20 despite him receiving a triple therapy based on an integrase strand transfer inhibitor. An HIV genotypic test while on dolutegravir/lamivudine/abacavir at weeks 12 and 20 disclosed the emergence of 184 M>V at population sequencing. Recent studies have reported a significant delay in the initial virological response and a poorer immune recovery in non-B HIV subtype, particularly subtype F, present in our case and typical of our region [43,44]. The emergence of integrase resistance HIV mutations during initial triple-drug therapy containing dolutegravir or bictegravir has been described with the accumulation of factors associated with poorer prognosis including a non-B subtype of HIV-1, a high viral load and low CD4+ T-cell count, poor adherence to ART, coinfections influencing drug levels, hospital admission and drug interactions [45,46,47,48,49,50]. Our patient gathered all these risk factors, but integrase resistance mutations did not emerge despite the selection of 184 M>V in the reverse transcriptase. Ultradeep sequencing was not available, therefore the potential emergence of minority variants in integrase could not be ruled out. On the other hand, the patient was on directly observed ART during long-term periods of hospital admission, but a poor adherence after discharging home or decreased drug absorption due to intestinal involvement from MAC infection cannot be excluded.

To the best of our knowledge, this is the first case report of concomitant association of SARS-CoV-2, MAC and *P. jirovecii* infection in a newly diagnosed HIV-1 infection with severe immune depletion, in the setting of an IRIS. Despite a very low CD4+ T-cell count, high HIV viremia, and multiple severe opportunistic infections, a persistent Alpha SARS-CoV-2 infection was not associated with significant intra-host evolution with the emergence of relevant mutations. The unexpected asymptomatic course of COVID-19 was associated with comparable spike-specific T-cell response levels, although enriched in IL-2, to HIV-negative matched controls. Thus, despite the spike-specific T-cell response protected this individual against a severe COVID-19 outcome, the increased immunological exhaustion marker (PD1) levels in his response might have favoured SARS-CoV-2 persistence.

## Data Availability

All data and materials used in the analysis have been available to any researcher for purposes of reproducing or extending the analysis.
